# Schwere und letale Vergiftungsfälle im Rettungsdienst – eine 5-Jahres-Analyse des Giftinformationszentrum-Nord

**DOI:** 10.1007/s00101-025-01544-3

**Published:** 2025-06-13

**Authors:** Markus Roessler, Sarah Anja Müller, Andreas Schaper

**Affiliations:** 1 Medizinische Fakultät und Universitätsklinikum OWL, Klinikum Bielefeld, Universitätszentrum für Anästhesiologie und Notfallmedizin, Universität Bielefeld, Teutoburger Straße 50, 33604 Bielefeld, Deutschland; 2 Zentrale Notaufnahme, Klinikum Wolfsburg, Wolfsburg, Deutschland; 3 Giftinformationszentrum Nord der Länder Bremen, Hamburg, Niedersachsen und Schleswig-Holstein, Universitätsmedizin Göttingen, Göttingen, Deutschland

**Keywords:** Intoxikationen, Antidottherapie, Giftnotrufzentralen, Poisoning Severity Score, ATC-Klassifikation, Intoxications, Antidote therapy, Poison control centers, Poisoning severity score, ATC classification

## Abstract

**Hintergrund:**

Vergiftungsnotfälle machen 3–7 % aller Rettungsdiensteinsätze aus. Welche Toxine zu schweren und letalen Vergiftungen führen, wurde in Deutschland bislang nicht genau analysiert.

**Methode:**

In dieser Untersuchung wurden alle über einen 5‑Jahres-Zeitraum vom Rettungsdienst dem GIZ-Nord gemeldeten Vergiftungen, die einen schweren oder tödlichen Verlauf hatten, analysiert. Die Vergiftungsschwere wurde mittels Poisoning Severity Score (PSS) [4] klassifiziert, und die ursächlichen Noxen wurden nach dem ATC-System [6] zugeordnet.

**Ergebnisse:**

Von 8528 Anfragen waren in 259 Fällen die Intoxikationen schwer, in 14 Fällen letal. Am häufigsten betroffen waren 20- bis 49-Jährige. In 221 (81 %) dieser Fälle erfolgte die Intoxikation in suizidaler Absicht und am häufigsten im häuslichen Umfeld. In 199 Fällen waren Arzneimittel, die mehrheitlich von Frauen (60,8 %) eingenommen wurden, ursächlich. Die häufigsten Substanzen waren Medikamente mit Wirkungen auf das Nervensystem, am zweithäufigsten, aber nur in 16 Fällen, Medikamente mit Wirkung auf das kardiovaskuläre System. In 29 Fällen (10,6 %) waren chemische Produkte auslösendes Agens, und nur bei 14 Fällen (5,1 %) waren es Drogen. Tödliche Verläufe wurden bei 14 Patienten (5,1 %) erfasst. Die meisten dieser Patienten wurden leblos aufgefunden und konnten nicht wiederbelebt werden. Drei Patienten wurden noch in eine Klinik transportiert, starben aber dort. Bei den letalen Intoxikationen waren prinzipiell geeignete Antidota nur in 3 Fällen verfügbar; bei diesen waren Wiederbelebungsmaßnahmen aber nicht mehr indiziert oder nicht erfolgreich.

**Schlussfolgerung:**

Diese Untersuchung zeigt, dass sich die meisten schweren und tödlichen Intoxikationen im häuslichen Umfeld in suizidaler Absicht mithilfe von Arzneimitteln ereignen. Aufgrund der möglichen Zahl unterschiedlicher Substanzen und mit Blick auf die Kombinationsmöglichkeiten ist es Rettungsdienstpersonal nicht möglich, alle Effekte und Stoffinteraktionen einschätzen zu können. Bei einem frühzeitigen Kontakt zu einer GIZ kann diese durch ihren Zugriff auf große Datenbanken wichtige Empfehlungen hinsichtlich symptomatischer oder weiterführender Therapie und zu geeigneten Zielekrankenhäusern geben.

**Graphic abstract:**

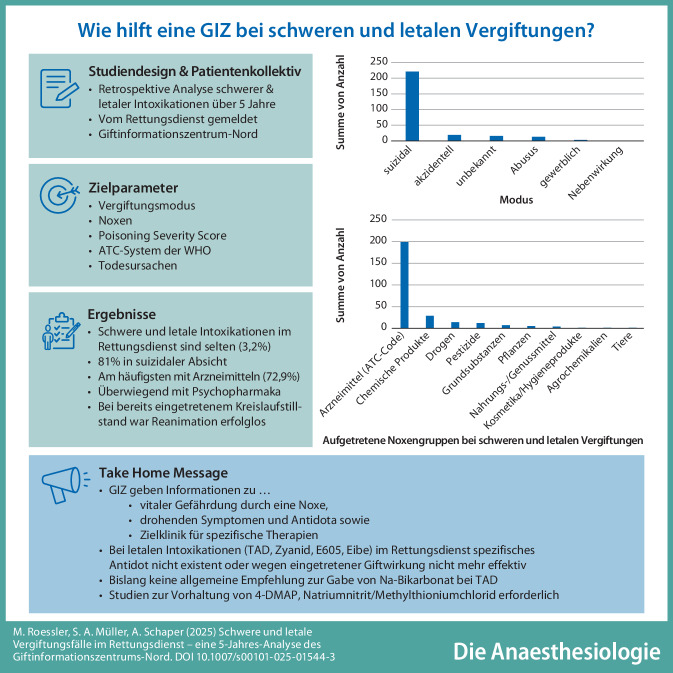

## Abstract (en)

**Background:**

Cases of poisoning account for 3–7% of all emergency medical services (EMS) calls. At present it is not known which toxins are responsible for severe and fatal poisoning in Germany.

**Methods:**

In this study, all cases of poisoning with a severe or fatal course reported to the Poisoning Information Center (GIZ) North by the emergency services over a 5-year period were analyzed. The severity of poisoning was classified using the poisoning severity score (PSS) [4] and the causative noxious agents were assigned according to the anatomical therapeutic chemical (ATC) classification system [6].

**Results:**

In 259 cases out of 8528 consultations by the EMS the poisoning was severe and in 14 cases it was fatal. Most commonly involved were patients between 20 and 49 years. In 221 (81%) of these cases the poisoning was with suicidal intent and most often at home. In 199 cases the cause was pharmaceuticals, mostly taken by women (60.8%). Most substances were pharmaceuticals with effects on the central nervous system, the second most frequent but only in 16 cases were drugs with effects on the cardiovascular system. Chemical products accounted for 29 (10.6%) and drugs for 14 (5.1%) of the cases. The outcome was fatal in 14 (5.1%) patients. Most of these patients were already dead when found and resuscitation efforts were unsuccessful. Of the patients three were transported to hospital but were dead on arrival. In fatal intoxications suitable specific antidotes were principally available only in three cases but resuscitation efforts were either no longer indicated or were unsuccessful.

**Conclusion:**

This study shows that most severe and fatal poisonings occur at home with suicidal intent using pharmaceuticals. Due to the possible number of different substances and the possible combinations, it is not possible for EMS personnel to assess all effects and substance interactions. If contacted early, due to their access to large data banks poisoning information centers are able to provide important recommendations with respect to symptomatic and further treatment as well as to suitable target hospitals.

## Hintergrund

Bei etwa 3–7 % aller Rettungsdiensteinsätze werden Patienten mit Intoxikationen behandelt [1]. Aufgrund ihres klinisch uneinheitlichen Bildes und der vielfältigen Möglichkeiten sind Intoxikationen für Mitarbeiter des Rettungsdienstes eine Herausforderung. Bisher gibt es aber nur wenige systematische Analysen zu Vergiftungen im Rettungsdienst. Eine retrospektive Studie der Christophorus Flugrettung in Österreich analysierte die Charakteristika von 599 intoxikierten Patienten in den Jahren 2006–2012. Dabei hatten 3,0 % der Patienten eine Vergiftung, die zu einem Kreislaufstillstand geführt hat [2]. Eine Studie der „Rettung Chur“ hat 169 Intoxikationen in der präklinischen Notfallversorgung für ein algorithmusbasiertes Vorgehen analysiert [3]. Hier fanden die Autoren keine letalen und nur 4 (2,4 %) vital bedrohliche Vergiftungen. In Deutschland wurden bisher keine genauen Fallanalysen zu schweren und letalen Vergiftungen im Rettungsdienst publiziert. Ziel dieser Untersuchung ist daher, die schweren und letalen Vergiftungsfälle aus dem Rettungsdienst, die dem Giftinformationszentrum(GIZ)-Nord der Universitätsmedizin Göttingen (UMG) im Zeitraum von 2014 bis 2018 gemeldet wurden, systematisch zu analysieren, um eine Übersicht über die Patientencharakteristika, die gemeldeten Noxen, die Vergiftungsumstände und Therapieansätze zu erhalten.

Diese retrospektive Untersuchung erfolgte mit Zustimmung der lokalen Ethikkommission der Universitätsmedizin Göttingen (Antrag Nr. 18/1/23).

## Material und Methoden

Das Giftinformationszentrum-Nord (GIZ-Nord) ist 24/7/365 für die telefonische Beratung bei Vergiftungsnotfällen für die Länder Bremen, Hamburg, Niedersachsen und Schleswig-Holstein zuständig. Es wurde am 01.01.1996 gegründet und erhält täglich etwa 150 bis 200 Anfragen; im Jahr werden knapp 50.000 Anfragen von Ärzt*innen, medizinischem und nichtmedizinischem Personal sowie Laien aus der Bevölkerung beantwortet [4]. Hierfür werden eine Präsenzbibliothek und mehrere i. d. R. nicht öffentlich zugängliche Datenbanken (z. B. www.toxinz.com, www.micromedexsolutions.com) genutzt. Alle beratungsrelevanten Dokumente (Fachinformationen, Sicherheitsdatenblätter, toxikologische Publikationen, insgesamt ca. 5 Mio. Dokumente) sind in der institutsinternen Datenbank GIZ-Index gespeichert. Bei jeder telefonischen Beratung wird ein Protokoll angelegt. Auch diese Beratungsprotokolle (ca. 900.000) sind in der Datenbank hinterlegt. Folgende Empfehlungen werden ausgesprochen: „Patient bleibt zu Hause“, „Vorstellung beim Arzt“, „Vorstellung beim Arzt beim Auftreten von Symptomen“, „stationäre Überwachung“ oder „intensivmedizinische Überwachung“. Darüberhinausgehende Empfehlungen, wie die Gabe eines Antidots oder spezifische Therapiemaßnahmen, werden auf dem Beratungsprotokoll als Freitext dokumentiert. Diese Daten bilden die Grundlage zur Beratung für künftige Anfragen und werden fortlaufend aktualisiert. Informationen über die Noxen, den Schweregrad der Intoxikation, die Symptomatik und Therapieempfehlungen werden dabei gespeichert. Die Vergiftungen werden nach dem Poisoning Severity Score (PSS) klassifiziert ([4, 5]; Tab. 1). Die Klassifizierung erfolgt an 12 möglichen Organen bzw. Organsystemen: GI-Trakt, respiratorisches System, ZNS, kardiovaskuläres System, metabolisches System, Leber, Niere, Blut, Muskulatur sowie lokale Effekte an der Haut, den Augen oder durch Bisse/Stiche. Die Symptome bzw. die Zeichen einer Vergiftung werden anhand einer 5‑stufigen Skala bewertet: 0 = keine, 1 = schwach, 2 = moderat, 3 = schwer, 4 = letal, wobei das schwerste Symptom den Schweregrad bestimmt. Die Einstufung erfolgt durch Ärzte, die den Fachtitel Humantoxikologe führen bzw. sich in der Weiterbildung hierzu befinden. Das GIZ-Nord benutzt zudem das anatomisch-therapeutisch-chemische Klassifikationssystem (ATC-System oder ATC-Code) der Weltgesundheitsorganisation (WHO) [https://www.whocc.no/atc_ddd_index/ und https://www.bfarm.de/DE/Kodiersysteme/Klassifikationen/ATC/_node.html;jsessionid=E8AE965C4C180948D2D356140CB0C322.intranet262], um Vergiftungen mit Arzneimitteln allgemeingültig und international gut nachvollziehbar und vergleichbar kategorisieren zu können. Insgesamt besteht das ATC-System aus 5 Ebenen. Die erste Ebene umfasst 14 Hauptgruppen (Tab. 2), z. B. Gruppe A: Medikamente mit Wirkung auf das alimentäre System und den Stoffwechsel, Gruppe M: Medikamente mit Wirkung auf das muskuloskeletale System und Gruppe N: Medikamente mit Wirkung auf das Nervensystem [6]. Diese Hauptgruppen lassen sich in weitere Untergruppen einteilen, z. B. Gruppe N für die Untergruppen Psychoanaleptika, Psycholeptika oder Antiepileptika [6].

**Tab. 1:** Schweregradeinteilung der Vergiftungsnotfälle nach dem Poisoning Severity Score (PSS). (Adaptiert nach Persson et al. [5] und Schaper [4])

Schwergrad	Symptome	Erklärung
0	Symptomlos	Keine Vergiftung
1	Leicht	Geringgradige, vorübergehende Symptome: Atemwegsreizung, leichte Luftnot, leichte Bronchospastik, vereinzelte Extrasystolen, passagere leichte Hypo- und Hypertension, Müdigkeit, Schwindel, Unruhe, Übelkeit, Erbrechen, Schluckstörung, Verbrennung 1. Grades
2	Mittelschwer	Symptome, die in der Regel einer ärztlichen Behandlung bedürfen: anhaltender Hustenreiz, Bronchospasmus, Luftnot, Stridor, Sauerstoffbedarf, Sinusbradykardie (HF < 60/min) oder -tachykardie (HF ca. 140–180/min), häufige Extrasystolen, Vorhofflimmern, Hypo- und Hypertension, Somnolenz, Verwirrtheit, anhaltendes Erbrechen, Durchfall
3	Schwer	Lebensbedrohliche Symptome: z. B. manifeste respiratorische Insuffizienz, schwere Sinusbradykardie (HF < 40/min) oder -tachykardie (HF > 180/min), lebensbedrohliche Herzrhythmusstörungen, AV-Block 3. Grades, Schock, hypertensive Krise, tiefes Koma, Status epilepticus, massive GI-Blutung, Verbrennung 2. Grades > 50 % KOF
4	Tödlich	Tödliche Vergiftung

*HF* Herzfrequenz, *KOF* Körperoberfläche

**Tab. 2:** Anatomisch-therapeutisch-chemische Klassifikationssystem (ATC-System oder ATC-Code) der Weltgesundheitsorganisation (WHO), adaptiert nach https://www.whocc.no/atc_ddd_index/ und https://www.bfarm.de/DE/Kodiersysteme/Klassifikationen/ATC/_node.html;jsessionid=E8AE965C4C180948D2D356140CB0C322.intranet262 und https://www.wido.de/publikationen-produkte/arzneimittel-klassifikation/ [6]

ATC-Code, Ebene 1	Anatomische Hauptgruppen
A	Alimentäres System und Stoffwechsel
B	Blut und blutbildende Organe
C	Herz-Kreislauf-System
D	Haut
G	Urogenitalsystem und Sexualhormone
H	Hormonpräparate zur systemischen Anwendung (außer Sexualhormone und Insulin)
J	Antiinfektiöse Substanzen zur systemischen Anwendung
L	Antineoplastische und immunmodulierende Substanzen
M	Muskuloskeletales System
N	Nervensystem
P	Antiparasitäre Substanzen und Insektizide
R	Atmungsorgane
S	Sinnesorgane
V	Verschiedenes

Zur Analyse der schweren und letalen Vergiftungsnotfälle wurden die Daten aller Protokolle von Vergiftungen, die nach dem PSS aufgrund des Verlaufes als schwer bzw. tödlich eingestuft worden waren, in der Datenbank des GIZ-Nord identifiziert und weiter analysiert, insbesondere mit Blick auf die Noxen und deren Zuordnung nach dem ATC-System.

## Ergebnisse

**Abb. 1:**
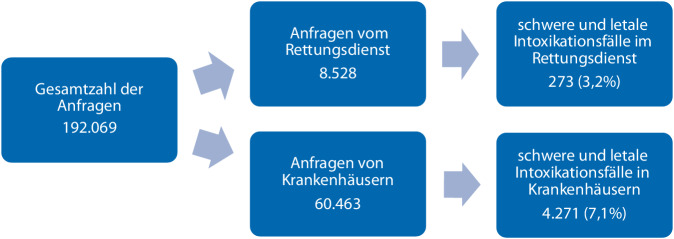
Anfragen an das GIZ-Nord vom 01.01.2014 bis 31.12.2018

Das GIZ-Nord wurde vom 01.01.2014 bis 31.12.2018 insgesamt 192.069-mal wegen einer Intoxikation kontaktiert. Von diesen Anfragen wurden 8528 (4,4 %) durch den Rettungsdienst gestellt. Davon wurden 259 Fälle als schwere und 14 letale Intoxikationen nach dem Poisoning Severity Score (PSS) [5] eingestuft und konnten weiter analysiert werden (Abb. 1). Die übrigen Anfragen erfolgten u. a. durch Krankenhäuser, Arztpraxen, Kindergärten und Privatpersonen.

Tab. 3 weist die Patientencharakteristika in Bezug auf Geschlecht, Alter, Vergiftungsmodus und Noxengruppen aus. Mit 108 Fällen (39,6 %) waren die 20- bis 49-Jährigen von schweren und tödlichen Intoxikationsfällen im Rettungsdienst im Vergleich zu anderen Altersgruppen am häufigsten betroffen. Unter den Kindern und Jugendlichen waren die 15- bis 18-Jährigen mit 16 Fällen (6,2 %) die größte Gruppe. Daneben waren 7 Kinder (2,6 %) im Alter unter 14 Jahre von Intoxikationen betroffen.

**Tab. 3:** Patientencharakteristika (*n* = 273)

	*n*	%
**Gesamt**	**273**	**100**
Männer	128	46,9
Frauen	139	50,9
Unbekannt	6	2,2
**Altersklassen**		
Kinder, Jugendliche	24	8,8
Erwachsene	244	89,4
Unbekannt	5	1,8
**Vergiftungsmodus**		
Suizidal	221	81
Akzidentell	19	7
Abusus	13	4,8
Gewerblich	3	1,1
Nebenwirkung	1	0,4
Unbekannt	16	5,9
**Noxengruppen**		
Arzneimittel (ATC-Klassifikation)	199	72,9
*ATC‑A (Medikamente mit Wirkung auf das alimentäre System und den Stoffwechsel)*	5	1,8
*ATC‑B (Medikamente mit Wirkung auf Blut- und blutbildende Organe)*	2	0,7
*ATC‑C (Medikamente mit Wirkung auf das kardiovaskuläre System)*	16	5,9
*ATC‑D (Dermatika)*	1	0,4
*ATC‑J (Antiinfektive Medikamente zum systemischen Gebrauch)*	2	0,7
*ATC‑L (Antineoplastische und immunmodulierende Medikamente)*	2	0,7
*ATC‑M (Medikamente mit Wirkung auf das muskuloskeletale System)*	3	1,1
*ATC‑N (Medikamente mit Wirkung auf das Nervensystem)*	154	56,4
*ATC‑P (Antiparasitäre Mittel, Insektizide, Repellents)*	1	0,4
*ATC‑R (Medikamente mit Wirkung auf den Respirationstrakt)*	13	4,8
Chemische Produkte	29	10,6
Drogen	14	5,1
Pestizide	12	4,4
Grundsubstanzen	7	2,6
Pflanzen	5	1,8
Nahrungs‑/Genussmittel	4	1,5
Kosmetika/Hygieneprodukte	1	0,4
Agrochemikalien	1	0,4
Tiere	1	0,4

**Abb. 2:**
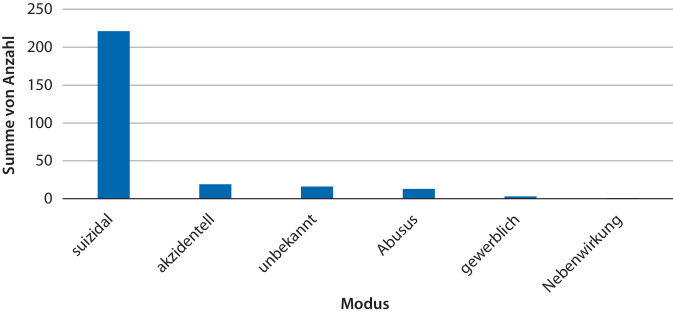
Vergiftungsmodi

**Abb. 3:**
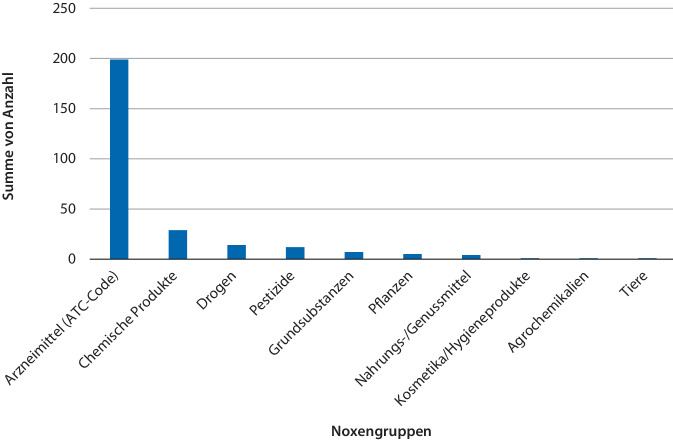
Noxengruppen

Intoxikationen in suizidaler Absicht waren mit 221 (81 %) der Fälle am häufigsten (Abb. 2). 19 Fälle (7,0 %) waren akzidentell. Bei 5 der 7 Kinder war es zu einer akzidentellen Vergiftung mit chemischen Produkten gekommen. In einem Fall wurde ein Medikament, in einem anderen Fall wurden 8 Dosen eines Energy-Drinks geschluckt. Bei insgesamt 13 Intoxikationen (4,8 %) handelte es sich um einen Abusus. Von den 22 akzidentellen Expositionen (8,1 %) ereigneten sich 3 Expositionsfälle (1,1 %) am Arbeitsplatz. Ein Fall (0,4 %) war auf Nebenwirkungen zurückzuführen. Der Modus war bei 16 (5,9 %) der gemeldeten Giftnotfälle unbekannt. In der mit 199 Fällen (72,9 %) größten Noxengruppe handelte es sich um Vergiftungen mit Arzneimitteln (Abb. 3). Hiervon erfolgten 185 (67,8 %) in suizidaler Absicht, 3 (1,1 %) aufgrund eines Medikamentenabusus, 2 (0,7 %) akzidentell und in einem Fall (0,4 %) aufgrund einer Nebenwirkung. Bei 8 Arzneimittelintoxikationen (2,9 %) war der Modus unbekannt. Chemische Produkte waren in 29 Fällen (10,6 %) ursächlich, 14 Expositionen (5,1 %) standen im Zusammenhang mit Drogen, Pestizide wurden in 12 Fällen (4,4 %) verwendet. In 7 Fällen (2,6 %) handelte es sich bei den Noxen um Grundsubstanzen (Stoffe ohne definiertes Anwendungsgebiet, z. B. Schwefelwasserstoff, Ameisensäure, Ammoniaklösung, Butandiol, Kaliumhexacyanoferrat). Zu einer Intoxikation mit Pflanzen kam es in 5 Fällen (1,8 %), mit Nahrungs- und Genussmitteln in 4 Fällen (1,5 %). In jeweils einem Fall (0,4 %) handelte es sich um Kosmetika/Hygieneprodukte, Agrochemikalien und ein Tier (Wald-Klapperschlange, lat. *Crotalus horridus atricaudatus*).

## Arzneimittelintoxikationen

Von insgesamt 199 Arzneimittelintoxikationen (72,9 %) waren mehrheitlich Frauen betroffen (121 weibliche Patienten, 75 männliche Patienten, 3 Patienten mit unbekanntem Geschlecht). Mit Blick auf die Medikamentenuntergruppen gemäß ATC-Code (Tab. 2) war die größte Gruppe mit 154 Fällen (56,4 %) der Hauptgruppe N des ATC-Codes mit Wirkung auf das Nervensystem zuzuordnen (Tab. 3 und 4). Bei 82 dieser Fälle waren Psychoanaleptika, zu denen trizyklische Antidepressiva, selektive Serotonin-Wiederaufnahmehemmer und Serotonin-Noradrenalin-Wiederaufnahmehemmer gehören, ursächlich und stellten damit die größte Untergruppe dar. Bei der zweitgrößten Untergruppe handelte es sich mit 44 Intoxikationsfällen um die Gruppe der Psycholeptika, zu denen Benzodiazepine sowie typische oder atypische Neuroleptika gehören. In 18 Fällen traten Intoxikationen mit Analgetika auf, welche damit die drittgrößte Untergruppe darstellen. Antiepileptika waren 9‑mal vertreten, und in einem Fall vergiftete sich ein Patient mit dem Anti-Parkinson-Mittel Memantin (Tab. 3 und 4).

**Tab. 4:** Schwere und tödliche Intoxikationen mit Arzneimitteln (*n* = 199) nach ATC-Klassifikation. (Modifiziert nach Fricke et al. [6])

ATC Code	Hauptgruppe	Untergruppe	Präparate	*n*
A	Medikamente mit Wirkung auf das alimentäre System und den Stoffwechsel	Laxanzien	Bisacodyl	1
		Antidiabetika	Metformin, Insuline	4
B	Medikamente mit Wirkung auf Blut- und blutbildende Organe	Antianämika	Eisen(II)-glycin-sulfat, Eisen(II)-sulfat	2
C	Medikamente mit Wirkung auf das kardiovaskuläre System	Herzmittel	Digoxin	1
		Diuretika	Torasemid	1
		β‑Blocker	Bisoprolol, Metoprolol	5
		Kalziumkanalblocker	Amlodipin, Verapamil	7
		ACE-Hemmer	Ramipril	2
D	Dermatika	Psoriasis-Mittel	Methoxsalen	1
J	Antiinfektive Medikamente zum systemischen Gebrauch	Antibiotika	Sulfamethoxazol, Trimethoprim	1
		Mittel gegen Mykobakterien	Rifampicin	1
L	Antineoplastische und immunmodulierende Medikamente	Antineoplastika	Methotrexat	2
M	Medikamente mit Wirkung auf das muskuloskeletale System	Antiphlogistika, Antirheumatika	Ibuprofen	2
		Muskelrelaxanzien	Methocarbamol	1
N	Medikamente mit Wirkung auf das Nervensystem	Analgetika	Acetylsalicylsäure, Tilidin, Morphin, Oxycodon, Hydromorphon, Tramadol, Paracetamol, Tapentadol	18
		Antiepileptika	Clonazepam, Phenobarbital, Valproinsäure, Eslicarbazepin	9
		Anti-Parkinson-Mittel	Memantin	1
		Psycholeptika	Alprazolam, Chlorprothixen, Lithium, Clozapin, Diazepam, Levomepromazin, Lorazepam, Melperon, Oxazepam, Pipamperon, Quetiapin, Prothipendyl, Risperidon, Olanzapin	44
		Psychoanaleptika	Amfebutamon, Amitriptylin, Citalopram, Clomipramin, Coffein, Doxepin, Escitalopram, Fluvoxamin, Imipramin, Mirtazapin, Opipramol, Moclobemid, Tranylcypromin, Trimipramin, Venlafaxin	82
P	Antiparasitäre Mittel, Insektizide, Repellents	Antiprotozoenmittel	Hydroxychloroquin	1
R	Medikamente mit Wirkung auf den Respirationstrakt	Antihistaminika zum system. Gebrauch	Cetirizin, Diphenhydramin, Promethazin*	13

*ACE* Angiotensinkonversionsenzym

* Neuroleptikum, entsprechend ATC-Code R eingeordnet

Mit 16 Fällen (5,9 %) bildeten Medikamente der ATC-Hauptgruppe C mit Wirkung auf das kardiovaskuläre System zweitgrößte Gruppe. Am dritthäufigsten waren mit 13 Fällen (4,8 %) Medikamente der ATC-Hauptgruppe R mit Wirkung auf den Respirationstrakt ursächlich (Tab. 3 und 4).

In der vorliegenden Untersuchung waren bei den durch Medikamente verursachten Todesfällen folgende Präparate dokumentiert: Koffeintabletten, Prothipendyl, Opipramol, Metformin, Bisoprolol und Mirtazapin sowie Melperon und Alprazolam (Tab. 4 und 5). In 2 von 5 Todesfällen war ein tri- oder tetrazyklisches Antidepressivum für einen vergiftungsbedingten Todesfall im Rettungsdienst verantwortlich (Tab. 4 und 5).

**Tab. 5:** Tödliche Vergiftungsnotfälle im Rettungsdienst, die dem GIZ Nord gemeldet wurden, 2014–2018 (*n* = 14)

Alter (Jahre)	Noxe	Fallbeschreibung	GIZ-Beratung
35 J	Koffein	Orale Aufnahme von ca. 8–9 g Koffein in Tablettenform, Modus unbekannt Primär erfolgreiche Reanimation bei Kammerflimmern, 48 h später verstorben	Ab 5 g potenziell letal, Erläuterung der Symptomatik, Hinweis auf spezielle therapeutische Maßnahmen wie Gabe von Aktivkohle und Möglichkeit der Hämodialyse, kein Antidot
40 J	Prothipendyl	Pat. wurde tot aufgefunden, vermutlich Aufnahme einer unklaren Menge Prothipendyl, 40-mg-Tabletten, in suizidaler Absicht	Anruf bei GIZ mit der Frage, ob Noxe todesursächlich sein kann. Keine Beratung zur Therapie mehr möglich
46 J	Opipramol	Orale Ingestion von 10 g Opipramol in suizidaler Absicht, in Notaufnahme hypodynamischer Kreislaufstillstand bei Asystolie	Dosis potenziell letal, Symptomatik erläutert, Aktivkohlegabe nach Intubation und Endoskopie zur Entfernung bei Retardpräparat empfohlen, mögliche Therapieoptionen Hämoperfusion [19] und va-ECMO diskutiert
53 J	Eibe (Taxin)	Orale Aufnahme von 100 ml Eibennadeltee in suizidaler Absicht, Dosis unklar, Erbrechen, primär erfolglose Reanimation bei therapierefraktärem Kammerflimmern	Symptomatik erläutert, Therapieoptionen va-ECMO und Digitalisantikörper [20] besprochen
54 J	Eibe (Taxin)	Orale Aufnahme von Beeren und Nadeln unbekannter Dosis in suizidaler Absicht. Primär erfolglose Reanimation bei hyperdynamischem Kreislaufstillstand	Symptomatik erläutert, Therapieoptionen wie VA-ECMO und Digitalisantikörper [20] besprochen
56 J	Metformin, Bisoprolol, Mirtazapin	Orale Mischintoxikation mit max. 100 Tbl. Mirtazapin, 15 mg; max. 120 Tbl. Metformin, 500 mg; max. 100 Tbl. Bisoprolol, 2,5 mg, in suizidaler Absicht, mit sicheren Todeszeichen aufgefunden	Zur Information der GIZ. Keine Beratung zur Therapie mehr möglich
68 J	Melperon, Alprazolam	Orale Mischintoxikation mit 30 Tbl. Melperon, 25 mg, und 30 Tbl. Alprazolam, mit sicheren Todeszeichen aufgefunden	Anruf bei GIZ mit der Frage, ob Dosis der Noxe todesursächlich sein kann
71 J	E 605 (Parathion)	Aufnahme von (fraglich 0,5 l) E 605 in suizidaler Absicht, mit sicheren Todeszeichen aufgefunden	Anruf bei GIZ, um Gefährdung des Ersthelfers (Laienreanimation) zu klären
78 J	Klarspüler	Akzidentelle Aufnahme einer unklaren Menge Klarspüler, akute respiratorische Insuffizienz bei chemischer Pneumonitis 3 Wochen nach dem Ereignis an Lungenversagen verstorben	Gefährdung durch Aspiration, Gabe von Entschäumer empfohlen
84 J	Duschgel	Akzidentelle Aufnahme von einer halben Flasche Duschgel bei vorbestehendem hirnorganischen Psychosyndrom im Pflegeheim Akute respiratorische Insuffizienz bei chemischer Pneumonitis, an Lungenversagen verstorben	Symptomatische Therapie (Intubation & Beatmung), Gabe von Entschäumer empfohlen
85 J	E 605 (Parathion)	Aufnahme von E 605 unklarer Dosis p.o. in suizidaler Absicht, frustrane Reanimation	Antidot Atropin, Dosierung nach Herzfrequenz und Ausmaß der Bronchorrhö
> 18 J	Laminatreiniger	Pat. wurde tot aufgefunden, vermutlich Aufnahme unbekannter Menge Pocoline®-Laminatreiniger	Anruf bei GIZ mit der Frage, ob Noxe todesursächlich sein kann. Keine Beratung zur Therapie mehr möglich
> 18 J	Kaliumhexacyanoferrat(III) (Cyanid)	Orale Ingestion unklarer Dosis Kaliumhexacyanoferrat(III) und 37,5 %iger Schwefelsäure in suizidaler Absicht, primär erfolglose Reanimation	Hinweis, dass Blausäure entstehen kann [21]; 250 mg 4‑DMAP und 100 mg/kgKG Natriumthiosulfat
> 18 J	Parathionprodukt	Orale Aufnahme einer unbekannten Menge Parathion in suizidaler Absicht, hypodynamischer Kreislaufstillstand bei Asystolie, primär erfolglose Reanimation	Anruf bei GIZ mit der Frage, ob Noxe todesursächlich sein kann, und ob Eigengefährdung möglich. Keine Beratung zur Therapie mehr möglich

## Chemische Produkte und Pestizide

Bei insgesamt 29 (10,6 %) Intoxikationen mit chemischen Produkten waren mehrheitlich Männer betroffen (19 männliche und 8 weibliche Patienten, in 2 Fällen war das Geschlecht unbekannt). Insgesamt 14 Fälle waren akzidentell bedingt und 11 Fälle in suizidaler Absicht. Bei einem Patienten (0,4 %) kam es aufgrund eines Abusus zur Intoxikation, bei einem weiteren (0,4 %) am Arbeitsplatz. In 3 Fällen (1,1 %) war der Modus bei Intoxikationen mit chemischen Produkten unbekannt. Bei den Vergiftungsnotfällen mit Pestiziden erfolgten 11 Fälle in suizidaler Absicht (4,0 %) und ein weiterer Fall (0,4 %) akzidentell.

## Drogen

In dem hier untersuchten Kollektiv hatten 14 (5,1 %) Patienten (12 männlich, 2 weiblich) schwere Intoxikationen, die auf Drogen zurückzuführen waren. Am häufigsten handelte es sich um Amphetaminderivate (*n* = 5), gefolgt von Cannabinoiden (*n* = 3), Organonitraten (*n* = 1), Liquid Ecstasy (*n* = 1), Kokain (*n* = 1), Lysergsäurediethylamid (LSD, *n* = 1) und nicht näher bezeichneten Drogen (*n* = 1).

## Vergiftungsort

Der Ort der gemeldeten schweren Vergiftungen war in absteigender Häufigkeit im häuslichen Umfeld (*n* = 255/273), unbekannt (*n* = 8/273), am Arbeitsplatz (*n* = 3/273), im Alten‑/Pflegeheim (*n* = 3/273), in der Justizvollzugsanstalt (*n* = 2/273), im Obdachlosenheim (*n* = 1/273) und im Krankenhaus (*n* = 1/273).

## Letal verlaufende Intoxikationen

Insgesamt 14 der 273 (5,1 %) gemeldeten schweren Intoxikationen nahmen einen letalen Verlauf und sind in Tab. 5 zusammengefasst.

## Diskussion

In der hier vorgelegten retrospektiven Untersuchung präklinischer Daten wurden in einem Zeitraum von 5 Jahren insgesamt 273 gemeldete schwere und tödliche Vergiftungsnotfälle aus dem Rettungsdienst analysiert.

Die meisten der schweren Vergiftungen (81 %) erfolgten in suizidaler Absicht, meist durch Einnahme von Arzneimitteln. Der häufigste Vergiftungsort war in dieser Untersuchung mit 93,4 % das häusliche private Umfeld, was gut zu publizierten Daten aus der Schweiz und Norwegen passt [3, 8]. Im Gegensatz zu anderen Publikationen [2, 3, 8, 9], die neben Arzneimitteln in erster Linie Alkohol und Opioide als am häufigsten anzutreffende Noxen benennen, scheinen Letztere in der vorliegenden Untersuchung unterrepräsentiert – mutmaßlich, weil auch der präklinische Umgang mit schwer alkoholisierten oder opiatintoxikierten Patienten gut bekannt und routiniert ist und der Giftnotruf seltener deswegen kontaktiert wird.

In dieser Untersuchung waren schwere Vergiftungen bei Kindern und Jugendlichen relativ selten (8,8 %) und ereigneten sich überwiegend akzidentell und ohne tödliche Verläufe in dieser Kohorte. Beide Erkenntnisse sind bereits vorbeschrieben [10]. Brockstedt berichtete von einer Letalität < 0,5 % auch bei Vergiftungen in suizidaler Absicht bei Jugendlichen [10]. Dennoch stellen Vergiftungsnotfälle bei Kindern und Jugendlichen eine nicht zu unterschätzende Gefahr dar; eine genaue Anamnese ist unerlässlich [4, 10].

Am häufigsten (72,9 %) erfolgte eine Vergiftung mit Arzneimitteln, wobei in 126 von 199 Fällen Psychoanaleptika oder Psycholeptika eingenommen wurden; diese waren damit für 63,2 % der Arzneimittelintoxikationen bzw. 46,2 % aller Vergiftungen verantwortlich. Ob diese Medikamente zuvor verordnet waren, wird in den Beratungsprotokollen i. d. R. nicht erfasst, zum einen, da Angaben hierzu nicht überprüfbar sind, und zum anderen, da dies für die Beratung hinsichtlich der Toxizität zumeist nicht relevant ist. Nach Paschen et al. [9] sind 10–13 % aller intoxikationsbedingten Todesfälle durch trizyklische und tetrazyklische Antidepressiva bedingt [9]. In den Vereinigten Staaten von Amerika und in Europa zählen Vergiftungen mit trizyklischen Antidepressiva zu den häufigsten Medikamentenintoxikationen und stellen die häufigste Todesursache mit rezeptpflichtigen Substanzen dar [11]. In dieser Untersuchung waren sogar 21,2 % der tödlichen Intoxikationen auf die Einnahme von Psycholeptika bzw. Psychoanaleptika zurückzuführen. Bei den primär nichttödlichen dieser Vergiftungen wurde stets wurde empfohlen, die Vitalfunktionen kontinuierlich zu überwachen. Nur vereinzelt – wenn die Zeit von Einnahme bis zum Erstkontakt < 60 min betrug und der Patient bewusstseinsklar war, wurde die Einnahme von 1 g/kgKG medizinischer Kohle empfohlen. Die Gabe von Natriumbikarbonat als Antidot bei Vergiftungen mit TAD, SSRI, atypischen oder typischen Neuroleptika wird empfohlen, wenn eine QRS-Verbreiterung im EKG gesehen wird [14–16]. Dieser Empfehlung folgen aber nicht alle GIZ, und auch bei den Vergiftungsfällen in dieser Studie wurde die Gabe von Natriumbikarbonat nicht empfohlen.

Nur 14 schwere und keine tödliche Intoxikation waren auf die Einnahme von Drogen (Organonitrate, Cannabinoide, Liquid Ecstasy, LSD, Amphetamin- oder Kokainderivate) und nichtklassifizierte Substanzen zurückzuführen. Hierbei muss aber bedacht werden, dass die GIZ nicht wahrscheinlich nicht kontaktiert wurde, wenn es sich um einen eindeutigen Todesfall durch eine Überdosierung, z. B. durch ein Opioid, gehandelt hat.

In der vorliegenden Untersuchung verliefen 14 der 273 gemeldeten schweren Intoxikationsfälle (5,1 %) tödlich. Bezogen auf den analysierten 5‑Jahres-Zeitraum und die insgesamt 8825 Anfragen aus dem Rettungsdienst in dieser Zeit (Abb. 1) entspricht dies einem Anteil von 0,2 % aller Anfragen an das GIZ-Nord aus dem Rettungsdienst. Die meisten Patienten wurden tot oder reanimationspflichtig aufgefunden; in 3 Fällen konnten die Patienten noch lebend ins Krankenhaus transportiert werden und verstarben dort (Tab. 5).

Eine schnelle, möglichst zielgenaue Therapie sowie die optimale supportive Behandlung schon im Rettungsdienst tragen dazu bei, vergiftete Patienten bestmöglich zu versorgen. Auf arztbesetzten und z. T. auch auf nichtarztbesetzen Rettungsmitteln werden Antidota, die bei spezifischen Vergiftungen unmittelbar eingesetzt werden sollen, vorgehalten. Diese Antidota (Atropin, Aktivkohle, 4‑Dimethylaminophenol (4-DMAP), Naloxon, Toloniumchlorid) werden in der „Bremer Liste“ genannt [4, 10, 12, 13].

Die Auswahl dieser wenigen Antidota zeigt, dass bei Anfragen durch den Rettungsdienst primär Fragen nach einer potenziellen vitalen Gefährdung, nach einem ggf. unmittelbar wirksamen Antidot und einer geeigneten Zielklinik, die nicht selten unter Zeitdruck beantwortet werden sollen, im Vordergrund stehen. Bei Anfragen von Kliniken werden hingegen intensiver die Möglichkeiten der toxikologischen Diagnostik und von Therapieoptionen, die geeignet sind eine vitale Gefährdung erst gar nicht entstehen zu lassen (z. B. Paracetamol) oder mit denen Sekundärschädigungen vermieden werden können, erörtert.

Bei den letal verlaufenden Intoxikationen dieser untersuchten Kohorte waren nur in 3 Fällen grundsätzlich geeignete Antidota verfügbar (Atropin bei E605-Vergiftung, 4‑DMAP für Kaliumhexacyanoferrat(III)-Intoxikation). Zu den Therapien liegen jedoch hier keine systematischen Angaben vor.

Die vorliegende Analyse zeigt, dass eine Intoxikation mit Cyaniden extrem selten ist. Wie bei dem Fall in dieser Studie muss davon ausgegangen werden, dass der Notruf nicht mehr durch den suizidalen Patienten selbst erfolgt, sondern erst verzögert durch Dritte. Reanimationsbemühungen sind daher in solchen Fällen praktisch immer erfolglos. Auch wurde über einen Zeitraum von 5 Jahren keine Intoxikation mit einem Methämoglobinbildner erfasst. Vor diesem Hintergrund kann diskutiert werden, ob die grundsätzliche Vorhaltung von 4‑DMAP, das, ebenso wie Toluidinblau, häufig nicht verfügbar ist, bzw. die Alternativsubstanzen Natriumnitrit (Nithidote™) bzw. Methylthioniumchlorid (Proveblue®) auf arztbesetzten Rettungsmitteln sinnvoll ist.

In der Mehrzahl der Fälle wurde in dieser Studie aufgrund der eingesetzten Noxe keine spezifische Antidottherapie empfohlen und eine symptomatische Behandlung empfohlen.

Nicht nur, weil die Möglichkeiten einer Vergiftung vielfältig sind, sondern insbesondere, weil Medikamentenintoxikationen am häufigsten sind und diese oft als Mischintoxikation vorliegen, sollte stets frühzeitig und großzügig Kontakt zu einem der 7 Giftinformationszentren in Deutschland erfolgen, da nur diese in kurzer Zeit Zugriff auf große Datenbanken haben, mit deren Hilfe individuelle und spezifische Empfehlungen gegeben werden können.

Der Rettungsdienst kontaktiert den Giftnotruf in der Regel, um Antworten auf folgende Fragen zu erhalten: Ist eine Noxe potenziell gefährlich? Welche Symptome können sich während der Prähospitalzeit entwickeln? Müssen spezifische oder therapeutische Maßnahmen ergriffen werden, und gibt es ein spezifisches Antidot? Der Giftnotruf berät diesbezüglich und darüber hinaus, welche Zielklinik aufgrund verfügbarer Ressourcen (z. B. Gastroenterologie zur Endoskopie, ECMO) angesteuert werden sollte. Aufgrund der vielfältigen Recherchemöglichkeiten können die Giftnotrufzentralen die Toxizität einer Noxe einschätzen, auf mögliche und drohende Symptome hinweisen und ggf. die Gabe eines Antidots empfehlen. Schwer intoxikierte Patienten sollten i. d. R. auf Intensivstationen mit internistischem Schwerpunkt behandelt werden. Bei dialysierbaren Noxen (z. B. Lithium) sollte die Möglichkeit einer Hämodialyse gegeben sein [17].

## Limitationen

Trotz einer seit 1990 bestehenden gesetzlichen Meldepflicht für Vergiftungen und Verdachtsfälle werden aktuell in Deutschland Intoxikationen noch nicht systematisch registriert [7]. Das aktuelle Meldesystem lässt eine direkte Nachverfolgung oft nicht zu. Daher liegen für die hier ausgewerteten Fälle meist keine strukturierten Follow-up-Daten zur Therapie und zum Verlauf vor. Bei der Interpretation der Daten muss also auch stets berücksichtigt werden, dass es sich um präklinische Anfragen aus dem Rettungsdienst an das Giftinformationszentrum-Nord handelt, und nicht um eine registerbasierte Auswertung aller stattgefunden Intoxikationen in einer bestimmten Region zu einem definierten Zeitpunkt.

## Fazit

Jährlich betreffen ca. 4 % der Gesamtanfragen an das GIZ-Nord Vergiftungsnotfälle im Rettungsdienst. Die vorliegende retrospektive Untersuchung eines 5‑Jahres-Zeitraums zeigte, dass die meisten gemeldeten Intoxikationen

- erwachsene Patienten betrafen,
- oral zugeführt wurden,
- durch Arzneimittel als häufigster Noxe bedingt waren,
- in suizidaler Absicht zugefügt wurden und
- zu Hause passierten.

Der Kontakt zu einem Giftinformationszentrum sollte frühzeitig auch durch den Rettungsdienst erfolgen. So kann beispielsweise der Giftnotruf auch bei der Auswahl des anzusteuernden Krankenhauses wegweisend sein, falls eine spezifische apparative Ausstattung für die Therapie einer bestimmten Intoxikation notwendig sein sollte [18].

## Data Availability

Die zugrunde liegenden Daten der Arbeit werden auf begründete Anfrage zur Verfügung gestellt.
